# Draft Genome Sequence of *Metschnikowia australis* Strain UFMG-CM-Y6158, an Extremophile Marine Yeast Endemic to Antarctica

**DOI:** 10.1128/genomeA.00328-17

**Published:** 2017-05-18

**Authors:** Thiago M. Batista, Heron O. Hilário, Rennan G. Moreira, Carolina Furtado, Valéria M. Godinho, Luiz H. Rosa, Glória R. Franco, Carlos A. Rosa

**Affiliations:** a Departamento de Bioquímica e Imunologia, Universidade Federal de Minas Gerais, Belo Horizonte, Minas Gerais, Brazil; b Departamento de Microbiologia, Universidade Federal de Minas Gerais, Belo Horizonte, Minas Gerais, Brazil; c Instituto de Ciências Biológicas, Universidade Federal de Minas Gerais, Belo Horizonte, Minas Gerais, Brazil; d Instituto Nacional do Câncer, Rio de Janeiro, Brazil

## Abstract

Here we report the draft genome sequence of *Metschnikowia australis* strain UFMG-CM-Y6158, a yeast endemic to Antarctica. We isolated the strain from the marine seaweed *Acrosiphonia arcta* (*Chlorophyta*). The genome is 14.3 Mb long and contains 4,442 predicted protein-coding genes.

## GENOME ANNOUNCEMENT

The genus *Metschnikowia* comprises a clade consisting of approximately 81 species. The sexual life cycles of the members of this clade involve the formation of elongated asci containing two, often needle-shaped, spores (1). *M. australis* is a species endemic to Antarctica, and has been isolated from seawater, marine invertebrates, sponges, and macroalgae (2–6). Owing to the extremely cold environment of Antarctica, *M. australis* may have unique metabolic traits enabling it to survive under such stressful conditions; exploring these can help identify potential antifreeze compounds for biotechnological use.

We isolated *M. australis* strain UFMG-CM-Y6158 from a marine macroalgae, *Acrosiphonia arcta* (*Chlorophyta*), collected in Admiralty Bay of King George Island in Keller Peninsula, Antarctica (5). We cultivated the strain on marine agar (Himedia, India) at 10°C for 15 days, and the genomic DNA was isolated by phenol:chloroform (1:1) extraction. We assessed DNA quality by gel electrophoresis and determined its purity and quantity using both the NanoDrop 1000 UV-Vis spectrophotometer and the Qubit version 2.0 fluorometer with the Qubit dsDNA HS assay kit (Thermo Fisher Scientific). We used the Nextera XT DNA kit (Illumina) to construct paired-end libraries and assessed their quality using Bioanalyzer HS Assay (Agilent Technologies). Generated fragments with a mean length of 1,167 bp were sequenced using the Illumina MiSeq sequencer, whereas those with a mean length of 550 bp were sequenced using the Illumina HiSeq 2500 sequencer. The former generated 1,585,122 reads (2 × 301) with 35× coverage, while the latter generated 103,312,458 reads (2 × 101) with 745× coverage. We assembled the genome using SPAdes version 3.9.1 (7). The assembled draft genome consisted of 14,356,710 bp over 160 contigs (>505 bp) with a G+C content of 47.2%. The longest contig was 1,116,518 bp long, and the *N*_50_ contig length was 542,232 bp. CEGMA (8) analysis showed that the assembly was 95.9% complete, whereas analysis with BUSCO version 2 (9) using the *Saccharomycetales* lineage data set indicated 90.2% completeness based on the presence of conserved orthologous genes among species of the genus. We identified 4,442 protein-coding genes using MAKER2 (10). A sequence similarity search using the BLASTx tool in BLAST version 2.2.31+ (11) returned 4,348 protein matches (97.8%), with e-value ≤1e^−6^, against NCBI’s nonredundant database. We identified 249 tRNAs using tRNAscan-SE (12).

Using the OrthoVenn web platform (13), we compared *M. australis* protein-coding genes with those of two previously sequenced *Metschnikowia* genomes—*M. fructicola* and *M. bicuspidata*. The analysis showed that *M. australis* has a much shorter predicted proteome than that of *M. fructicola* (5,851 protein-coding genes) and *M. bicuspidata* (6,028 protein-coding genes). Additionally, we found six exclusive clusters of paralogous genes, of which four did not match any protein in the NCBI and UniProt-Swissprot databases. These results highlight the importance of investigating yeast endemic to Antarctica, such as *M. australis*, not only to identify novel genes associated with adaptation to extreme environments, but also for potential application in biotechnology.

### Accession number(s).

Data related to this whole-genome shotgun project have been deposited at DDBJ/ENA/GenBank under the accession number MVNQ00000000. The version described in this paper is the first version, MVNQ01000000.
